# Combined Tribenoside/Lidocaine Rectal Cream (Procto-Glyvenol^®^) Promotes Tissue Repair in a Preclinical Model of Acute Complicated Anal Fissure

**DOI:** 10.3390/ph19040612

**Published:** 2026-04-13

**Authors:** Ganna Zaychenko, Nazarii Kobyliak, Larysa Natrus, Maksym Tymofieiev, Patrizia Angelico, Stefano Biondi, Matteo Malinverno

**Affiliations:** 1 Endocrinology Department, Bogomolets National Medical University, 01601 Kyiv, Ukraine; n.kobyliak@csd.com.ua (N.K.); lnatrus777@gmail.com (L.N.); maksym.tymofieiev90@gmail.com (M.T.); 2 Medical Laboratory CSD LAB, 02000 Kyiv, Ukraine; 3 Preclinical Group, R&D, Recordati S.p.A., 20148 Milan, Italy; angelico.p@recordati.it (P.A.); blondi.s@recordati.it (S.B.)

**Keywords:** rectal cream, anal fissure, tribenoside/lidocaine, diltiazem, macroscopic assessment, morphological assessment, enzyme-linked immunosorbent assay, immunoblot analysis, molecular mechanism of action, effectiveness, Procto-Glyvenol

## Abstract

**Background/Objectives**: The objective of this study was to evaluate the efficacy of a rectal cream containing tribenoside and lidocaine (TL) in a rat model of anal fissure (AF) and to investigate the potential mechanisms of its therapeutic action compared with those of a standard care rectal cream containing 2% diltiazem (D). **Methods**: Treatment efficacy was assessed via macroscopic methods. The levels of the inflammatory factors IL-6 and IL-10 in the tissues were measured via ELISA. Histology assessment was performed with standard hematoxylin/eosin stain, Masson’s trichrome method and picrosirius stain. The levels of NF-κB, VEGF, TGF-beta 1, HIF-1α and E-cadherin were measured via densitometric immunoblot analysis. **Results**: The results of this study show that the medical product TL has therapeutic efficacy in a preclinical model of acute complicated AF, which is likely related to its complex composition. The severity of pathology in the TL group was significantly lower than that in the control pathology (CP) group on the eighth day of treatment and remained significantly lower on the 11th and 12th days. There was no statistically significant difference between the TL group and the CP group (*p* = 0.186 for IL-6 and *p* = 0.078 for IL-10). The efficacy of TL and D groups showed no statistically significant difference. At the end of the experiment, after 12 days of treatment, the level of the proinflammatory marker NF-κB in the CP group was greater than that in the intact control (IC) group. In turn, the NF-κB level in the TL group was lower than that in the CP group and significantly lower than that in the D group. Other important markers evaluated in this study demonstrated a similar tendency. The histopathological analysis showed that TL ointment promoted superior tissue repair, resulting in healthier anodermal architecture with minimal scarring and reduced fibrosis. **Conclusions**: This study confirms the potential for conducting further pharmacological studies of the mechanism of action and further clinical trials of the rectal cream TL, which has certain advantages in terms of effectiveness in a model of acute complicated AF.

## 1. Introduction

Anal fissure (AF) is a widespread disease (occurring in more than 2% of the adult population) that affects patients of both sexes, more often young and middle-aged people [1,2].

The negative impact of AF on the quality of life of patients due to the presence of unpleasant clinical symptoms, such as pain, bleeding, a tendency toward a chronic course and a significant prevalence, defines this pathology as an urgent medical and social problem that places a significant financial burden on both patients and the healthcare system [1,2].

According to the modern understanding of pathogenesis, pain causes spasm of the internal anal sphincter, which leads to impaired blood circulation in the AF area, hypoxia, and inflammation, which contribute to tissue damage on the one hand and inhibition of healing on the other hand, which in turn again causes pain, leading to a “vicious closed circle” [3,4,5,6,7].

According to the 2022 clinical guidelines of the American Society of Colon and Rectal Surgeons, for the treatment of AF, calcium channel blockers can be used as first-line drugs (level 1B), with diltiazem offering a strong safety profile. Calcium channel blockers offer similar efficacy to topical nitrates [8].

Tribenoside is an agent indicated for the local treatment of hemorrhoids. It decreases capillary permeability, enhances vascular tone, and exhibits anti-inflammatory properties. Its antagonistic action targets certain endogenous mediators involved in the development of inflammation and pain. Lidocaine is a local anesthetic that relieves itching, burning, and pain associated with hemorrhoids.

Diltiazem is a comparator that induces relaxation of vascular smooth muscle by blocking voltage-dependent L-type Ca^2+^ channels (CaV1.2), thereby reducing calcium influx into smooth muscle cells. The decrease in intracellular Ca^2+^ concentration inhibits the Ca^2+^-calmodulin–dependent activation of myosin light-chain kinase, resulting in reduced actin–myosin interaction and vascular smooth muscle relaxation [9].

According to the above information, investigations into the efficacy of tribenoside/lidocaine for the treatment of AF are of potential interest.

The objective of this study was to assess the efficacy of combined rectal cream tribenoside/lidocaine (TL) in a preclinical study on a model of acute complicated anal fissure (ACAF) in comparison with that of a standard care rectal cream with diltiazem for experimental justification of the possible use of this cream for the treatment of patients with AF.

## 2. Results

### 2.1. Macroscopic Assessment

**Figure 1:**
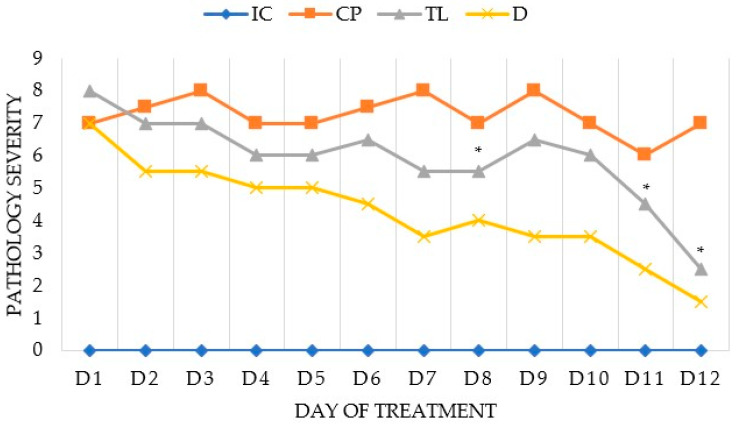
Macroscopic assessment of pathological severity, with comparison between the groups. * The difference is statistically significant compared with the CP group (*p* < 0.05).

From the first day of the experiment, animals with induced pathology exhibited increased swelling, hyperemia, local bleeding, purulent-necrotic processes, and anatomical defects. Pathology induction lasted for four days. The macroscopic severity of pathology in the three groups on the first day of treatment was approximately the same, ranging from 7 to 8 points (Figure 1). Statistical analysis was performed via the Wilcoxon rank-sum test (a distribution-free test for independent samples).

The severity of pathology in the control pathology (CP) group was significantly greater than that in the IC group starting from the first day of the experiment, and remained so until the end of the experiment. In the animals in the CP group, the severity of the pathology was consistently high, ranging from 7 to 8 points up to the 10th day of the experiment, and slightly decreasing to six points on the 11th day.

In the animals in the (TL) group, a significant decrease in the expression of the pathology was observed, starting from day 10. The severity of pathology in the (TL) group was significantly lower than that in the CP group on the eighth day of treatment, and remained significantly lower on the 11th and 12th days.

The severity of pathology in the diltiazem (D) group also gradually decreased with each day of treatment. Throughout the treatment period, there was no significant difference in pathology severity between the TL and D groups. The effectiveness of treatment in groups TL and D was similar.

### 2.2. Levels of Inflammatory Markers

**Figure 2:**
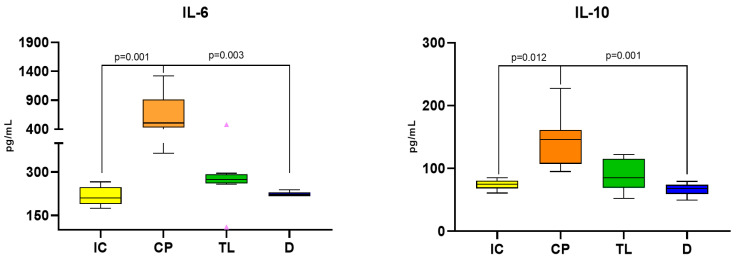
Concentration of IL-6 and IL-10 in tissue homogenates from the anodermal area of rats after 12 days of treatment in the AF model.

Evaluation of the levels of the IL-10 and IL-6 markers was conducted via ELISA. The concentration of the anti-inflammatory marker IL-10 in the tissue homogenate of the anodermal area of the rats at the end of the experiment was significantly greater in the CP group than in the IC group, which confirms the high concentration of inflammatory markers due to the significant inflammatory process in the untreated group (Figure 2).

The concentration of IL-6 in the tissue homogenate of the anodermal area of the rats after 12 days of treatment was significantly lower in the TL group than in the CP group (*p* = 0.001), which demonstrated a positive recovery trend in the TL group. The concentration of both IL-6 and IL-10 in the tissue homogenate of the anodermal area of the rats after 12 days of treatment was significantly lower in the D group than in the CP group (*p* = 0.003 and *p* = 0.001 respectively), which also demonstrated a positive recovery. There was no statistically significant difference between the TL group and the CP group (*p* = 0.186 for IL-6 and *p* = 0.078 for IL-10). The difference between the TL and D groups was not statistically significant for both cytokines (Figure 2).

### 2.3. Densitometric Immunoblot Analysis

**Figure 3:**
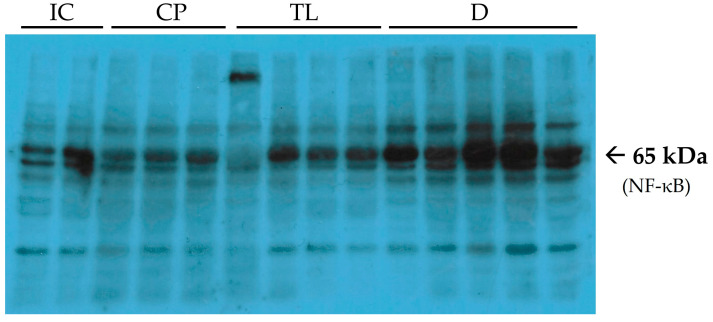
Immunoblots of nuclear factor kappa-b (NF-κB). Lanes 1–2 (from the left) are related to the IC group. Lanes 3–5 are related to the CP group. Lanes 6–9 are related to the TL group. Lanes 10–14 are related to the D group.

At the end of the experiment, after 12 days of treatment, the level of the proinflammatory marker NF-κB in the CP group was greater than that in the IC group. The treatment with TL significantly reduced the increased level of NF-Kb, while D had no effect (Figure 3).

**Figure 4:**
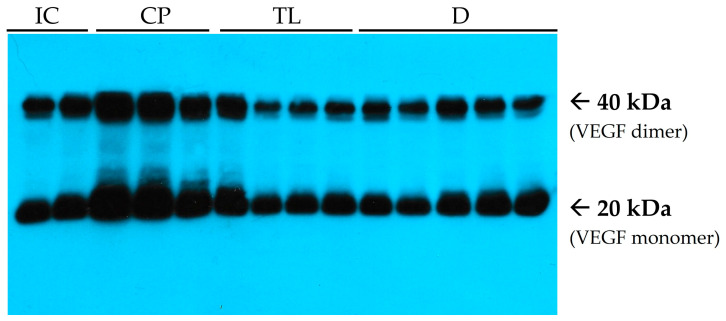
Immunoblots of vascular endothelial growth factor (VEGF). Lanes 1–2 (from the left) are related to the IC group. Lanes 3–5 are related to the CP group. Lanes 6–9 are related to the TL group. Lanes 10–14 are related to the D group.

At the end of the experiment, after 12 days of treatment, the level of VEGF in the TL group was significantly greater than that in the IC group. In turn, the VEGF levels in the TL and D group were significantly lower than that in the CP group with no statistically significant difference between the TL and D groups (Figure 4).

**Figure 5:**
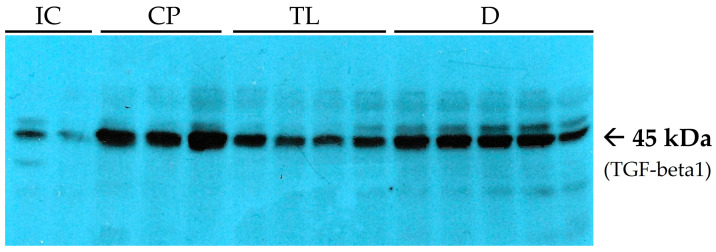
Immunoblots of transforming growth factor beta 1 (TGF-beta1). Lanes 1–2 (from the left) are related to the IC group. Lanes 3–5 are related to the CP group. Lanes 6–9 are related to the TL group. Lanes 10–14 are related to the D group.

The results revealed that, following tissue trauma, TGF-beta1 expression increased significantly and remained at a high level. In the context of AF healing, this indicates active tissue regeneration and stimulation of the fibrotic process. At the end of the 12 day treatment period, the level of the reparation marker TGF-beta1 in the CP group was significantly greater than that in the IC group (Table 1). This factor plays a key role in recruiting fibroblasts, stimulating collagen synthesis, and forming the extracellular matrix, which contributes to wound closure (Figure 5).

A decrease in TGF-beta1 expression may indicate the end of the active regeneration phase and a reduction in fibrotic activity. This could be a sign of normalized tissue remodeling and a decline in the inflammatory response. Among all the treatment groups, the TL group presented the most favorable results, with a reduction in the TGF-beta1 level. At the end of the experiment, the TGF-beta1 level in the TL group was significantly lower than that in both the CP and D groups (Figure 5).

Moreover, considering cytokine interplay, the decreased NF-kb and TGF-beta1 levels demonstrated in the TL group can be considered as reflecting a minimal residual proliferation wound healing phase, while in the CP and D groups, increased levels of NF-kb and TGF-beta1 lead to either self-sustainable inflammation or a prolonged proliferation phase, with possible excessive fibrosis.

**Figure 6:**
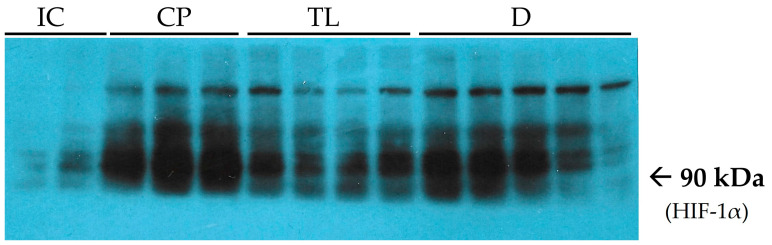
Immunoblots of hypoxia-inducible factor 1-alpha (HIF-1α). Lanes 1–2 (from the left) are related to the IC group. Lanes 3–5 are related to the CP group. Lanes 6–9 are related to the TL group. Lanes 10–14 are related to the D group.

At the end of the experiment, after 12 days of treatment, the level of the hypoxia marker HIF-1α in the CP group was significantly greater than that in the IC group (Table 1). A decrease in HIF-1α levels during treatment may indicate successful tissue repair and normalization of the oxygen balance. It can also reflect a reduction in inflammation and improved blood supply to the damaged area. At the end of the treatment period, the HIF-1α levels in the TL group were significantly lower than those in the CP group (Figure 6).

The increased HIF-1α in the CP group could indicate prolonged and extended inflammation and proliferation phases of wound healing, while a near twofold decrease in the HIF-1α level in the TL and D groups can be considered as a shift from the proliferation to the remodeling phase.

Near normal levels of VEGF in the TL and D groups support the idea that the inflammation phase of AF healing has already been passed by the end of the experiment, and the proliferation phase is near its completion. It should also be mentioned that VEGF secretion is promoted by HIF-1α, so a decrease in inflammation-related hypoxia leads to a decrease in vascular growth.

At the end of the experiment, after 12 days of treatment, the level of the adhesion marker E-cadherin in the CP group was significantly greater than that in the IC group (Table 1). In the early stages of wound healing, E-cadherin levels typically decrease, allowing cells to lose intercellular contact and gain motility. During the active regeneration phase, cells migrate to the injury site where extracellular matrix remodeling occurs. In the final stages, E-cadherin levels increase again, helping to restore the epithelial barrier and stabilize the tissue (Table 1).

**Figure 7:**
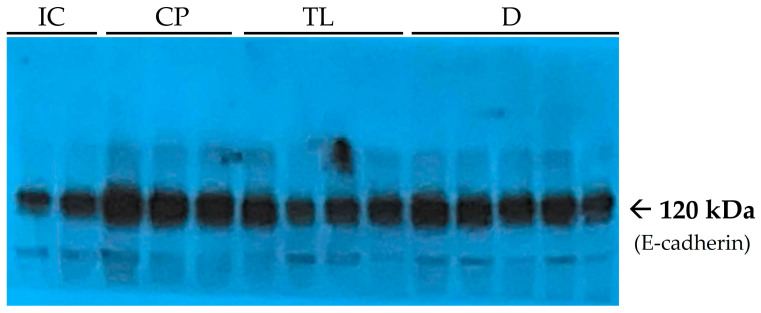
Immunoblots of E-cadherin. Lanes 1–2 (from the left) are related to the IC group. Lanes 3–5 are related to the CP group. Lanes 6–9 are related to the TL group. Lanes 10–14 are related to the D group.

At the end of the 12 day treatment period, E-cadherin levels in the TL group were significantly lower than those in the CP group (Table 1, Figure 7).

While both groups (TL and D) had comparable macroscopic healing at 12 days, the TL cream had a more pronounced effect on the modulation of certain molecular pathways, including inflammatory (NF-κB) and pro-fibrotic (TGF-β1) markers, which correspond to the quality of histology (mature collagen).

### 2.4. Morphological Assessment

An assessment of the significance of pathological changes and the therapeutic efficacy of treatment regimens was performed morphologically.

**Figure 8:**
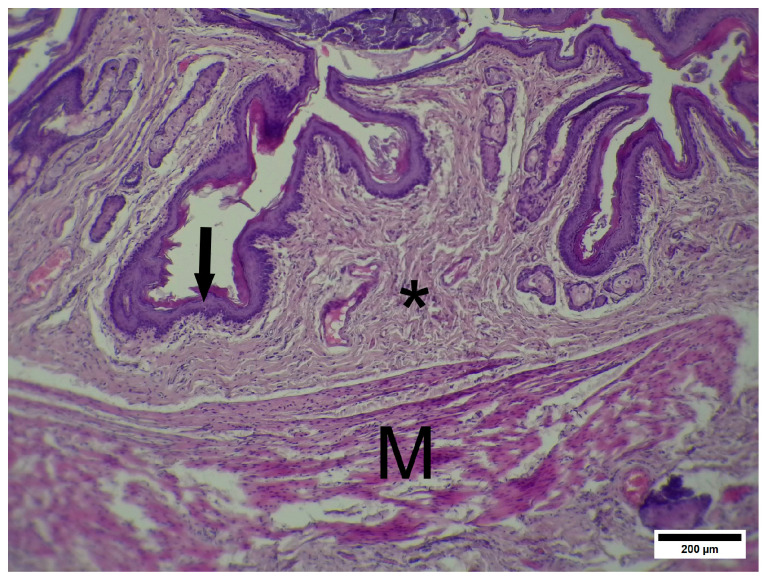
IC—anoderm zone. Squamous partially keratinized epithelium, submucosal connective tissue and the external anal sphincter. Arrow—striated epithelium. * Submucosal connective tissue, M—external sphincter striated muscle. Hematoxylin and eosin. Scale bar 200 µm.

The IC group presented a normal histological structure of the anoderm zone (Figure 8). The thickness of the epithelium is even among the parts of the channel. The submucosa is formed by loose connective tissue and contains submucosal blood vessels; some slices contain anal glands and perianal hair follicles. Below the submucosa, the anal sphincter is clearly seen as eosinophilic bands of striated skeletal muscle tissue. The most peripheric parts of the samples contained loose connective and adipose tissue, such as the adventitia and retroperitoneal space.

**Figure 9:**
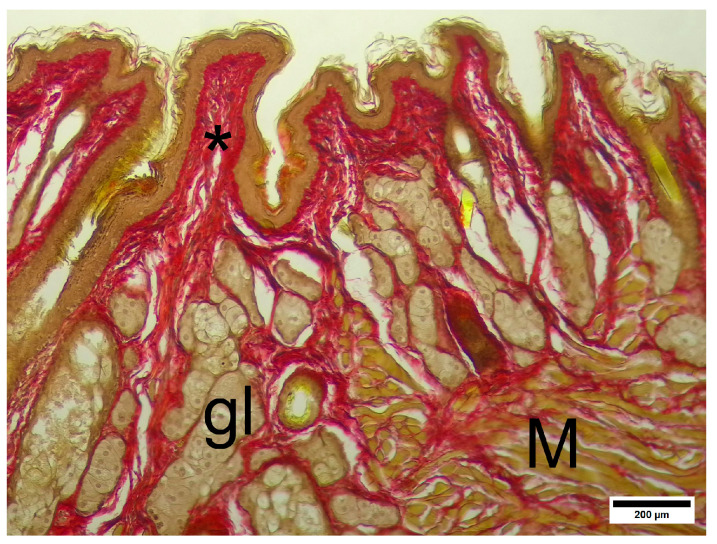
IC—anoderm zone. Epithelium and submucosa. * Submucosal connective tissue, gl—perianal glands, M—external sphincter striated muscle. Picrosirius strain. Unpolarized light. Scale bar 200 µm.

Picrosirius strain revealed (Figure 9) that the collagen bands in the submucosal connective tissue layer were composed primarily of type I collagen, which formed dense bands, stripes and dots in the submucosa and formed a fiber network below.

**Figure 10:**
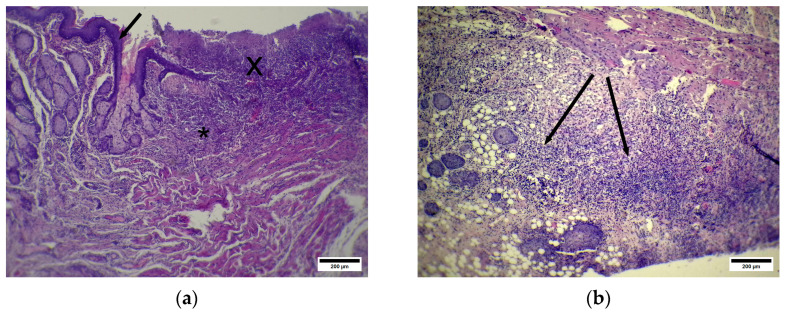
(**a**) CP—anoderm zone. Large defect zone without epithelization. Arrow—stratified squamous epithelium, X—debris at injury site. * WBC infiltration in submucosa. Hematoxylin and eosin. Scale bar 200 µm. (**b**) CP—anoderm zone. WBCs infiltrate the adventitia. Arrows—WBCs infiltrate the adventitia. Hematoxylin and eosin. Scale bar 200 µm.

The CP group demonstrated unequal anal fissure healing: in some animals, we observed large defects without epithelization (Figure 10a) with a defect zone infiltrated with WBCs.

The submucosa at the injured site contains a low number of glands and blood vessels. We also observed pronounced cellular infiltration in the adventitia at the fissure zone (below the muscular layer) (Figure 10b).

**Figure 11:**
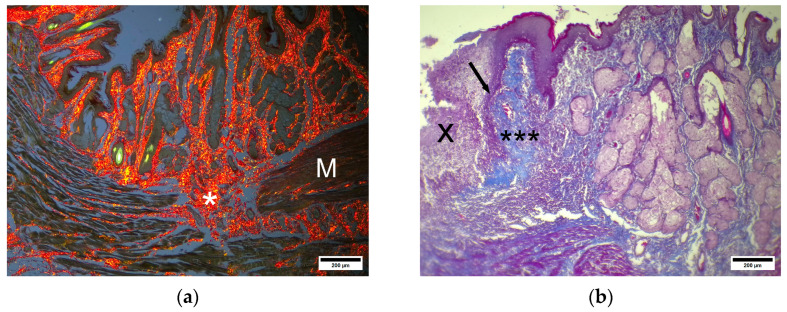
(**a**) CP—anoderm zone. * Newly formed dense scar tissue at disrupted sphincter site, M—external sphincter striated muscle. Picrosirius strain. Polarized light. Scale bar 200 µm. (**b**) (CP). Anoderm zone. X—debris at injury site. Arrow—newly formed epithelium. *** Newly formed connective tissue. Masson’s trichrome staining. Scale bar 200 µm.

Both Masson’s trichrome and picrosirius stains revealed scar formation in the case of complete defect healing. This scar extends from the submucosa to the muscular layer and is composed of chaotically oriented bands of collagen fibers that form wide avascular zones (Figure 11a).

**Figure 12:**
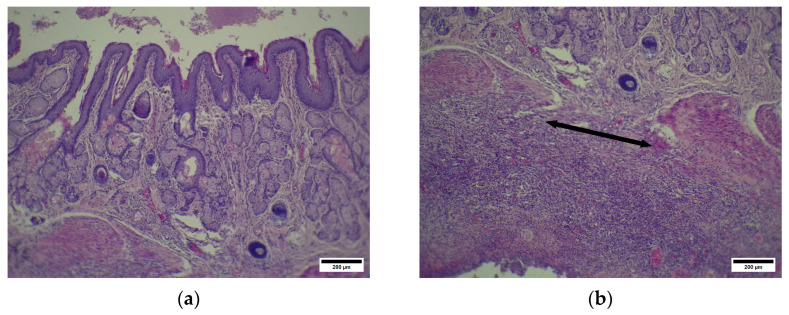
(**a**) TL—anoderm zone. Anal glands with eosinophilic content. Hematoxylin and eosin, scale bar 200 µm. (**b**) TL—anoderm zone. Disrupted sphincter with WBC infiltration (double-headed arrow). Hematoxylin and eosin, scale bar 200 µm.

In cases of incomplete, ongoing defect epithelization, the submucosa, which is located near the injury site, is greatly infiltrated by WBCs. This infiltration zone appears as a wide band with fuzzy outlines that separates the submucosa into different parts: the deeper, distal part, which contains a large number of dilated blood vessels, and the proximal part, which is situated in close proximity to the injury site, and contains wide fields of newly formed connective tissue that appear as bluish homogeneous masses with rare or even absent blood vessels (Figure 11b). The external sphincter is composed of thin striated muscular fibers with thick bluish bands of connective tissue between them. The TL group demonstrated signs of complete injury epithelization in the anoderm zone. The newly formed epithelium appears more basophilic, thicker, and composed of a greater number of layers (Figure 12a). Nevertheless, cells from superficial layers at the injury site contain basophilic granules and are covered by a superficial keratin layer. The submucosa at the anoderm zone contains many anal glands (Figure 12a), some of which have eosinophilic masses at the center. In the muscular layer, the sphincter appears completely disrupted (Figure 12b) and is underlined by the adventitia with cell infiltration.

**Figure 13:**
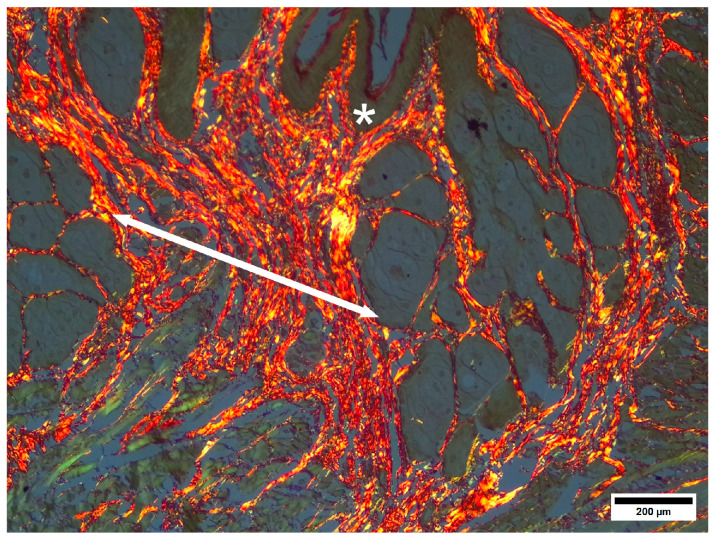
TL—anoderm zone. * Healed epithelium at injury site. Double-headed arrow—soft scar-oriented collagen fiber bands. Picrosirius strain. Polarized light. Scale bar 200 µm.

Picrosirius strain revealed that the submucosa at the injury site contains relatively thin but radially oriented collagen fibers with clear alignment. These fibers were intermitted by a substantial number of blood vessels with soft scar formation (Figure 13).

**Figure 14:**
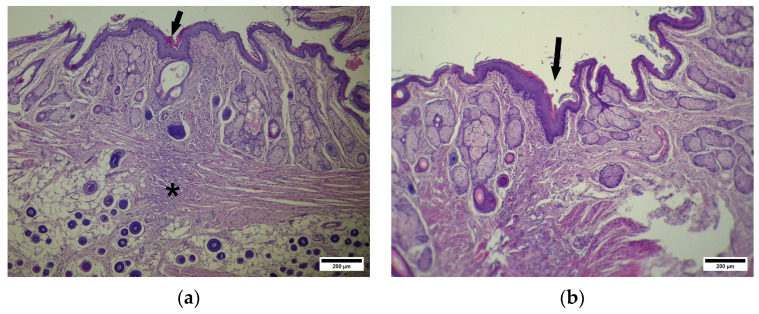
(**a**) D—anoderm zone. Anal glands with eosinophilic content. Arrow—epithelium at injury site. * Scar at the disrupted sphincter site. Hematoxylin and eosin, scale bar 200 µm. (**b**) D—anoderm zone. Anal glands with eosinophilic content. Arrow—epithelium at injury site. Hematoxylin and eosin, scale bar 200 µm.

The D group also demonstrated signs of complete epithelization of the injury site at the anoderm zone (Figure 14).

**Figure 15:**
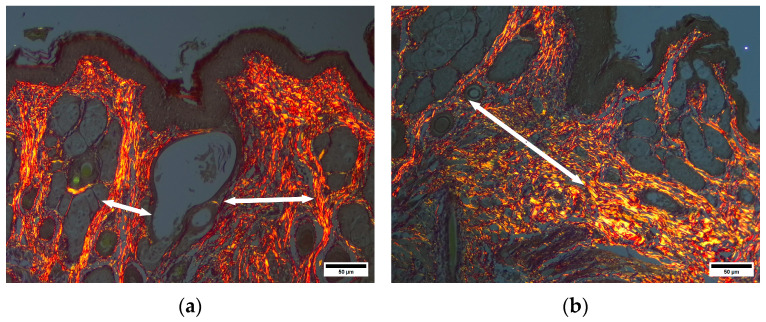
(**a**) D—anoderm zone. Arrows—scar tissue collagen fibers around injury site. Picrosirius strain. Polarized light. Scale bar 50 µm. (**b**) D—anoderm zone. Arrow—scar tissue collagen fibers around injury site. Picrosirius strain. Polarized light. Scale bar 50 µm.

Picrosirius strain revealed that the submucosal layer at the injury site contained thin narrow bands of collagen fibers. These bands were relatively ordered and aligned and were intermitted by a moderate number of newly formed blood vessels. The collagen bands at the injury site appeared thinner than those in neighboring areas and were situated more loosely (Figure 15).

Thus, the CP group demonstrated major incomplete and ongoing epithelization, with both prolonged inflammation and proliferation phases of AF healing. In contrast to this, the TL and D groups showed defect closure by epithelium and early signs of inflammation resolution. The main differences between the two treatment groups are as follows. Although both groups had closed fissure defect epithelium, in the TL group, it looks more mature in contrast with the D group. As for deep layers, both groups demonstrated defect healing with connective tissue growth, but the TL group had more mature collagen bands, while the D group had loose, immature collagen bands.

Such histopathological findings support results on cytokine levels and indicate that wound healing in the TL group occurs faster, including resolved hypoxia and proliferation phase completion with swift transition to the remodeling phase, while the D group continues ongoing scar tissue remodeling.

## 3. Discussion

AF and hemorrhoids are common anorectal disorders with a complex pathogenesis that has been significantly enriched by recent evidence. The course of AF is closely associated with internal anal sphincter spasm and inflammation, which explains the persistent discomfort and impaired quality of life in affected patients [10]. The pathological process primarily occurs in the anodermal area between the anus and perianal skin, where it can cause severe pain, swelling, bleeding, and purulent discharge. Without timely treatment, stromal framework disruption and architectural changes in the rectal mucosa occur, leading to complications such as fibrosis, fecal incontinence, bleeding, and eventually an increased risk of malignant transformation. For effective precision pharmacotherapy, it is important to understand the pathophysiology of AF, particularly the molecular-level alterations and identification of molecular targets that can guide pharmacological correction strategies [11].

Acute fissures—defined as those present for less than 6–8 weeks—can heal with topical pharmacological therapy (rectal creams, suppositories). Pharmacological sphincterotomy is recognized as the first-line treatment for chronic anal fissure [12]. It also has the potential to reduce overall treatment costs [8,13]. Owing to scarring and impaired blood flow, chronic fissures are often refractory to conservative therapy and require surgical intervention. Even after lateral internal sphincterotomy, topical therapy accelerates healing while avoiding the risk of incontinence [14,15,16,17].

The aim of our study was to evaluate the efficacy of a rectal ointment containing tribenoside and lidocaine (TL) in a rat model of anal fissure and to investigate the potential mechanisms of its therapeutic action compared with those of a rectal cream containing 2% diltiazem (D). The high efficacy of TL ointment in hemorrhoids has been well established in both preclinical and clinical studies [18,19,20], but its therapeutic effect on AF has not been previously studied.

In a model of acute complicated AF in rats, both preparations exhibited therapeutic effects beginning in the early days of treatment (based on macroscopic assessment of the anodermal area). Diltiazem cream demonstrated slightly greater efficacy on days 6–9, but by day 12, the healing efficacy of both products was comparable.

Analysis of the pharmacodynamics of TL ointment suggests that its effects are mediated through a complex influence on molecular markers involved in inflammation, cellular regeneration, and wound healing—key pathways in the pathogenesis of complicated AF.

The anti-inflammatory effect of TL ointment was accompanied by a decrease in the level of IL-6, a proinflammatory cytokine substantially elevated in the control pathology group. Excessive IL-6 production and dysregulated signaling can lead to inflammatory and autoimmune diseases as well as cancer [21]. In terms of IL-6 suppression, TL ointment was comparable to D cream.

NF-κB is an important transcription factor that plays a key role in regulating immune responses, inflammation, cell proliferation, apoptosis, and angiogenesis. It is particularly crucial in the inflammatory process, as it promotes the activation of genes responsible for inflammatory responses and suppresses apoptosis, potentially prolonging the inflammatory phase. The anti-inflammatory properties of TL ointment are further supported by its ability to reduce nuclear factor-κB (NF-κB), which drives the transcription of inflammatory mediators associated with pain, bleeding, and systemic manifestations [22]. Aberrant NF-κB activity disrupts tight epithelial junctions, increasing intestinal permeability [23]. Compared with D cream, TL ointment had a greater effect on NF-κB inhibition.

VEGF is a protein that plays a key role in angiogenesis, the formation of new blood vessels. It stimulates the growth and migration of endothelial cells lining vessel walls and promotes the development of new vasculatures during tissue regeneration. VEGF also helps establish alternative pathways for blood supply. Additionally, VEGF contributes to the dilation of existing blood vessels, enhances tissue perfusion, and increases the permeability of vascular walls, thereby facilitating metabolic exchange between the blood and surrounding tissues. TGF-beta1 is a strong cytokine that plays a key role in regulating various physiological processes, including wound repair. In healthy tissue, a certain basal level of this mediator is present, which is necessary to maintain normal cellular homeostasis. The pathophysiological role of TGF-beta1 is associated primarily with the development of fibrosis because of its ability to stimulate the transformation of fibroblasts into myofibroblasts, which actively synthesize collagen and other components of the extracellular matrix. Hyperactivation of this process may lead to excessive fibrosis, resulting in tissue stiffness and impaired function. The ability of TL ointment to regulate VEGF and TGF-β1 expression underlies its role in promoting epithelial repair while preventing excessive fibrosis. In fact, overactivation of these pathways, as observed in the control group, can lead to fibrotic remodeling, tissue rigidity, and functional impairment [24]. TL significantly reduced the overexpression of both VEGF and TGF- β1, with better efficacy than D.

Considering the pro- and anti-inflammatory roles of NF-kb and TGF-beta1, the low levels of these cytokines in the TL group can be associated with the near complete resolution of inflammation, whereas moderately increased NF-kb and TGF-beta1 in the D group can be explained by delayed, prolonged healing [25].

In normal tissue, the level of HIF-1alpha is minimal. This hypoxia-inducible factor plays a key role in regulating cellular adaptation to oxygen availability. Under physiological conditions, a balance between oxygen consumption and oxygen supply is maintained; thus, oxygen is present at a low basal level. An increase in HIF-1α levels in the case of AF may indicate local hypoxia and the progression of an inflammatory process. This may represent a tissue damage response that activates healing and regeneration mechanisms. TL also reduced HIF-1α, indicating improved oxygenation, restoration of tissue homeostasis, and reduced hypoxia in affected mucosal areas. Both treatments had comparable effects on this marker.

As was shown in studies on the wound healing expression of HIF-1alpha, VEGF is stage-specific and similarly increases during ongoing epithelization at the wound edge and then diminishes after wound closure. Our data on the decreased levels of HIF-1alpha and VEGF in the TL and D groups supports the conclusion about enhanced accelerated reepithelization under the treatment influence [26]. E-cadherin is a transmembrane protein that plays a key role in cell adhesion, helping maintain the integrity of epithelial tissues. Under normal conditions, it ensures intercellular contact between epithelial cells, supports their polarity and organization, and participates in signaling pathways that regulate cell proliferation and differentiation. A decrease in E-cadherin levels in the later stages of tissue repair may have different implications depending on the context of regeneration. If E-cadherin remains low, it may indicate chronic inflammation (associated with chronic pathological conditions, chronic wounds, etc.), which was not observed in this experiment. Under normal healing conditions, E-cadherin levels usually return to baseline levels as cells re-establish intercellular junctions and stabilize the epithelial barrier.

A significant decrease in E-cadherin levels may actually be a positive sign if accompanied by active tissue remodeling aimed at restoring homeostasis. In AF healing, E-cadherin supports epithelial barrier stabilization by enabling tight cell–cell junctions and tissue restoration. Therefore, its level tends to rise in response to epithelial injury as a compensatory mechanism.

However, as E-cadherin is a key adhesion molecule, its excessive expression may hinder epithelial-to-mesenchymal transition, limiting cell migration to the injury site. Elevated E-cadherin levels can reduce cellular plasticity, impairing migration, regeneration, and adaptation to new tissue conditions. If cells cannot effectively migrate and remodel tissue, excessive extracellular matrix accumulation may occur, promoting fibrotic changes. Also, E-cadherin levels nearly return to baseline upon treatment with TL, but not D; this adhesion molecule is essential for epithelial integrity and proper maturation of Paneth and goblet cells [27]. Treatment with TL greatly improved E-cadherin expression back to an acceptable level (Table 1); therefore, cell movement was facilitated which enabled the re-establishment of the epithelial barrier. Conversely, in the CP group, because the E-cadherin level was elevated, it was possibly indicative of a pathogenic compensatory overexpression which would impede healing from occurring.

Taken together, these data demonstrate that TL cream had a favorable effect on AF healing, outperforming D cream in terms of several key markers including signal molecules level and histopathology results.

The measured effects on IL-6, IL-10, NF-κB, VEGF, TGF-β1, HIF-1α, and E-cadherin are indeed associated with the therapeutic effect of TL, since the data were compared both with the CP group (untreated) and with the reference drug D. The composition of excipients in TL and D was identical; therefore, the effects of excipients can be excluded.

The observed biomarker improvements fully correlated with the morphological findings in the anorectal and rectal tissues, confirming the robust therapeutic effect of TL ointment in this complicated AF model.

Animals treated with TL ointment presented the most favorable outcomes, characterized by a mature epithelium at the injury site, the formation of soft, well-vascularized connective tissue, the restoration of epithelial–mesenchymal interactions, and the complete reestablishment of the epithelial barrier.

Our findings align with previous experimental data demonstrating that tribenoside (0.1 μM, 1 μM and 10 μM) increases fibroblast migration in vitro, enhances wound healing and re-epithelialization in rats, and exhibits antioxidant activity that may serve as an additional mechanism in AF repair [20].

The data we obtained regarding the positive effect of TL on the healing processes correlate with the results of TL studies in in vivo and in vitro model systems [26]. According to the approved prescribing information, the drug is registered for the treatment of hemorrhoids. Our study helped substantiate the potential for clinical research in a new indication—anal fissure.

Modern molecular markers of inflammation and regeneration were investigated for the first time, addressing the lack of data for a drug that has been used in clinical practice for many years.

In summary, TL ointment promoted superior tissue repair in the anal fissure model, resulting in healthier anodermal architecture with minimal scarring and reduced fibrosis. This translated into well-vascularized, soft connective tissue and the complete restoration of epithelial integrity, highlighting TL’s potential to support functional recovery and prevent long-term complications.

### Limitations

Sample size and power. There was a low sample size and consequently low statistical power, especially for the immunoblot data, so there may be differences that were not able to be demonstrated and therefore not identified as statistically different. Particularly, there may be significant differences in the IL-6 and IL-10 levels in the TL with regard to how they were expressed.

Use of acute complicated animal model. Since the research used an acute complicated model during the ACAF phase, the results from this research cannot be considered to apply directly to patients with chronic anal fissures because of the differences in the clinical presentation and possible different molecular profiles.

Exploratory nature of the mechanistic insights. Although the data from this study show different regulations of key pathways from the literature, it does not demonstrate the mechanism of the pathways directly through a causal chain. Future studies using specific inhibitors or knock-out models will need to be performed to corroborate the specific molecular targets of tribenoside.

The source of diltiazem cream and its bioavailability compared to a standard commercially available formulation may differ and serve as a confounding variable. TL does outperform the cream according to some key markers, including NF-κB and TGF-β1, but it is not true for every marker, such as IL-10, IL-6, and HIF-1α, where no difference was detected. Macroscopically, healing was comparable for both treatments at 12 days.

## 4. Materials and Methods

### 4.1. Overall Study Design

The study was performed on 28 nonlinear white male rats (200–240 g). Under local anesthesia (0.1 mL of a 0.5% solution of novocaine hydrochloride (Darnytsia: Kyiv, Ukraine)), anal fissure (AF) was modeled by dissecting the mucosa along the posterior wall of the anal canal with a scalpel (at the 6 o’clock position according to the conventional clock-face orientation, with the rat in the supine position), forming a defect—a linear wound measuring 5 × 2 × 1 mm. To enhance pathological manifestations (inflammation, edema), a phlogogenic agent—0.1 mL of a 2% aqueous formalin solution (Reagent: Dnipro, Ukraine)—was additionally injected into the submucosa of the posterior wall of the anal canal in the region of the transition of the skin into the anoderma. Treatment of the rats was initiated 96 h after AF induction [28].

All the animals were divided into four experimental groups. Group 1—intact control group (intact control; IC—six rats). Group 2—animals that were not treated with anal fissure (control pathology; CP—eight rats). Group 3—animals that were treated with the combined rectal cream tribenoside-lidocaine injected into the anal canal with an insulin syringe with a blunt needle at 0.3 mL once daily throughout the experiment—eight rats. Group 4—animals that were treated with 0.3 mL of the rectal cream “diltiazem 2%” injected into the anal canal with an insulin syringe with a blunt needle once daily throughout the experiment (group D—six rats). Pathology modeling lasted 4 days. The further treatment phase lasted 12 days. All manipulations with animals were carried out in accordance with the recommendations of the SEC of the Ministry of Health of Ukraine [29], the National “General ethical principles of animal experiments” [30], the Law of Ukraine No. 3447-IV of 21 February 2006 [31], as amended “On the protection of animals from cruel treatment” by the decision of the first national congress on bioethics [32] and “European Union Directive 2010/63/EU on the protection of animals used for scientific purposes” [33]. The study was started after the approval of the bioethical committee under Bogomolets National Medical University (protocol #185 dd. 27 May 2024).

The investigational medical product used was rectal cream tribenoside-lidocaine (serial number—PH3E72, expiry date—July 2026). For comparison, the medical product used was rectal cream “diltiazem 2%” (expiry date—December 2025) produced under laboratory conditions at the Scientific and Technological Complex “Institute of Single Crystals” of the National Academy of Sciences of Ukraine by Professor Lyapunov M.O.

Euthanasia of rats was performed by an overdose of sodium pentobarbital (150–200 mg/kg, intraperitoneally) in accordance with the recommendations of the AVMA Guidelines for the Euthanasia of Animals (2020) [34] and Directive 2010/63/EU [33]. Death was confirmed by the absence of respiration, cardiac activity, and the corneal reflex.

### 4.2. Macroscopic Assessment

The total macroscopic pathology process severity was assessed on a scale from 0 to 10 points on the basis of the sum of the five following parameters: severity of edema, hyperemia, local bleeding, purulent-necrotic processes, and anatomical defects (each parameter was assessed on a scale from 0 to 2 points).

### 4.3. Enzyme-Linked Immunosorbent Analysis

The levels of IL-10 and IL-6 in tissue were measured via immunoenzymatic analysis. The tissue samples were washed with precooled PBS buffer (0.01 M, pH = 7.4, Sigma-Aldrich: St. Louis, MO, USA) to remove blood residues, immediately frozen in liquid nitrogen and stored at −80 °C. For homogenate preparation, tissue samples were crushed with a pestle in porcelain crucibles with the addition of liquid nitrogen. After that, the homogenate was weighed, and chilled Lysis Buffer (FineTest: Wuhan, China) was added at a ratio of 1:9. The homogenates were further sonicated three times via a Sonoplus Mini 20 device (Bandelin Electronic GmbH: Berlin, Germany), and the tubes were kept on ice. The mixture was subsequently centrifuged (MicroMed: Kyiv, Ukraine) at 16,000× *g* for 15 min while cooling to 4 °C, after which the supernatant was collected for ELISA.

The concentrations of the markers were evaluated in the tissue lysates via a solid-phase enzyme-linked immunosorbent assay via the following kits according to their instructions. A photometer for microplates, HiPo MPP-96 (Biosan: Riga, Latvia), an automatic plate washer, 3D-IW8 Inteliwasher (Biosan: Riga, Latvia), and a thermal shaker for microplates, PST-60HL-4 (Biosan: Riga, Latvia), were used for the study. Data processing was carried out via QuantAssay 0.8.2.6 software (Biosan: Riga, Latvia).

### 4.4. Morphological Study

Materials from each animal (rectum with perianal skin) were harvested and fixed for 72 h in 4% formalin solution in PBS (pH 7.2; Sigma-Aldrich: St. Louis, MO, USA). For histological examination, we selected two sites: the anoderm zone and the anal canal transient zone. The material was dehydrated in isopropyl alcohol (Klebrig: Kyiv, Ukraine) and xylene (Starlab: Kyiv, Ukraine), embedded in Paraplast Plus (McCormick Leica Biosystems: Buffalo Grove, IL, USA), and sliced with a Sakura Accu-cut SRM-200 (Sakura: Tokyo, Japan) microtome. For general examination and epithelium assessment, we used standard hematoxylin and eosin stain with Mayer’s hematoxylin (Hematoxylin M, BioGnost: Zagreb, Croatia) and eosin. To assess connective and muscle tissue at the injury site, sections were strained via Masson’s trichrome method using Weigert’s iron hematoxylin, acid fuchsin and methyl blue. To assess collagen type I at the injury site, including connective tissue remodeling or scar formation, selected sections were stained with picrosirius using Weigert’s iron hematoxylin, picric acid, Sirius red (Direct red 80, Sigma-Aldrich: St. Louis, MO, USA), and observed via unpolarized and polarized light. The slices obtained were observed with a Micromed Evolution ES-4130 microscope (Micromed: Shenzhen, China). Pictures were processed via Image software (Ver. 1.50 NIH, National Institutes of Health: Bethesda, MD, USA).

To ensure the objectivity of the macroscopic pathological findings, all assessments were performed by two independent researchers who were strictly blinded to the treatment groups. To minimize subjectivity, any discrepancies between their individual scores were resolved through joint re-evaluation and discussion until a consensus was reached.

### 4.5. Immunoblot Analysis

For deeper analyses of molecular processes during the formation of a model pathology (AF) and the pharmacological effects of TL and D, immunoblot analyses were performed to determine the concentrations of indicative markers for AF. The following markers were assessed: NF-κB, VEGF, TGF-beta1, HIF-1α and E-cadherin. NF-κB (inflammatory marker) is an important transcription factor that plays a key role in regulating immune responses, inflammation, cell proliferation, apoptosis, and angiogenesis. Immunoblot analysis was performed according to the method developed by Towbin [35]. Proteins were transferred to the membrane via transfer buffer with the following composition: 25 mM Tris-HCl (pH 8.3, Thermo Fisher Scientific: Waltham, MA, USA), 0.192 M glycine (Brenntag: Essen, Germany), and 20% methanol (Sigma-Aldrich: St. Louis, MO, USA). The transfer voltage was 35–45 V at a current of 200–230 mA, and the transfer duration was 90–120 min. The membrane was washed with PBST. Before incubation with antibodies, a blocking procedure was performed at 37 °C for 60–90 min. After blocking, the membrane was incubated with antibodies against the target protein in PBST solution for 15–18 h at 4 °C. The blots were developed via enhanced chemiluminescence (ECL) in a dark room under weak red light. The membrane was treated with a solution of the following composition: 0.1 M Tris-HCl (pH 8.5, Thermo Fisher Scientific: Waltham, MA, USA), 0.25 M luminol solution (Sigma-Aldrich: St. Louis, MO, USA) in DMSO, 0.9 mM coumaric acid solution (Sigma-Aldrich: St. Louis, MO, USA) in DMSO, and 0.0035% hydrogen peroxide (Vishpha: Kyiv, Ukraine). After development, the films were air-dried and scanned for digital images, and densitometric analysis was conducted via the TotalLab program package (TLI20, Nonlinear, Inc.: Durham, NC, USA).

Statistical analysis was performed using the standard software SPSS version 20.0 (SPSS, Inc.: Chicago, IL, USA) and GraphPad Prism, version 6.0 (GraphPad Software, Inc.: La Jolla, CA, USA). Variables are presented as the mean and standard deviation of the mean (M ± SD). The IL-6 and IL-10 are represented graphically in the form of a box chart. The upper and lower bars correspond to the 25th and 75th quartiles, respectively, and the line passing through the middle of the square corresponds to the median value (Me). The differences among groups were assessed using the Kruskal–Wallis H test, followed by Dwass–Steel–Critchlow–Fligner post hoc tests for pairwise comparisons. Differences between the compared groups were considered statistically significant at *p* < 0.05. Multiple comparison adjustment using the Bonferroni test was applied to the *p*-values but did not make any impact on the interpretation of the results.

## Figures and Tables

**Table 1:** Densitometric immunoblot analysis of the groups after 12 days of treatment (mean ± standard deviation). * Statistically significant difference compared with the IC group, *p* < 0.05. ^#^ Indicates a statistically significant difference compared with the CP group, *p* < 0.05. ^†^ Indicates a statistically significant difference compared with group (D), *p* < 0.05.

Study Group	Average Value in the Group and Standard Deviation (A.U.). Mean ± Standard Deviation.				
	Inflammatory Marker	Regeneration Marker		Marker of Ischemia/Hypoxia	Adhesion Marker
	NF-kb	VEGF	TGF-Beta1	HIF-1α	E-Cadherin
IC	0.35 ± 0.25 (n = 2)	1.00 ± 0.18 (n = 2)	0.34 ± 0.14 (n = 2)	0.64 ± 0.25 (n = 2)	1.13 ± 0.44 (n = 2)
—	2.41 ± 2.84 (n = 3)	2.48 ± 0.58 * (n = 3)	3.94 ± 1.00 * (n = 3)	5.71 ± 0.35 * (n = 3)	3.14 ± 0.52 * (n = 3)
TL	0.78 ± 0.47 ^†^ (n = 4)	1.03 ± 0.49 ^#^ (n = 4)	0.87 ± 0.17 *^#†^ (n = 4)	2.31 ± 0.49 *^#^ (n = 4)	1.27 ± 0.81 ^#^ (n = 4)
D	2.78 ± 0.76 * (n = 5)	1.20 ± 0.15 (n = 5)	1.83 ± 0.58 *^#^ (n = 5)	2.59 ± 1.52 ^#^ (n = 5)	2.20 ± 0.53 (n = 5)

## Data Availability

The original contributions presented in this study are included in the article. Further inquiries can be directed to the corresponding authors.

## Abbreviations

The following abbreviations are used in this manuscript:

AF	Anal fissure
IL	Interleukin
ELISA	Enzyme-linked immunosorbent assay
ACAF	Acute complicated anal fissure
IC	Intact control
CP	Control pathology
TL	Tribenoside-lidocaine
D	Diltiazem
A.U.	Arbitrary units
NF-kb	Nuclear factor kappa-light-chain-enhancer of activated B cells
VEGF	Vascular endothelial growth factor
TGF-beta1	Transforming Growth Factor beta 1
HIF-1α	Hypoxia-Inducible Factor 1-alpha
E-cadherin	Epithelial cadherin
kDa	Kilodalton
